# Enhancing ZnO Efficiency: Humic Substances as Natural Carriers and Stabilizers for ZnO Nanoparticles for Sustainable Agriculture

**DOI:** 10.3390/nano16181183

**Published:** 2026-09-19

**Authors:** Tianyi Shen, Gleb N. Trishkin, Maria G. Chernysheva, Natalia A. Kulikova, Ivan V. Mikheev, Grigorii R. Chermashentsev, Sergey V. Maksimov, Gennadii A. Badun

**Affiliations:** 1 Department of Chemistry, Shenzhen MSU-BIT University, Shenzhen 518172, China; 2 Department of Chemistry, Lomonosov Moscow State University, Moscow 119234, Russia; trishkin.gleb1@gmail.com (G.N.T.); mikheev.ivan@gmail.com (I.V.M.); chermashentsev96@mail.ru (G.R.C.); irber@yandex.ru (S.V.M.); badunga@my.msu.ru (G.A.B.); 3 Department of Soil Science, Lomonosov Moscow State University, Moscow 119234, Russia; kulikova-msu@yandex.ru

**Keywords:** ZnO nanoparticles, humic acid-coated ZnO nanoparticles, Zinc uptake by plants

## Abstract

Zinc is an essential micronutrient in agricultural systems, fulfilling vital roles in both plant physiology and modern fertilizer formulations. Despite its importance, zinc deficiency has emerged as one of the most prevalent micronutrient limitations in global agriculture. Humic acids (HA) can serve as effective transporters of trace elements for crops, including in foliar fertilization. Our investigation addressed two key aspects: (1) the synthesis of HA-stabilized ZnO nanoparticles, and (2) the assessment of nanoparticle efficacy as zinc sources for foliar fertilization. ZnO and HA-coated ZnO nanoparticles were synthesized via a chemical deposition method and characterized by FTIR, XRD, DLS, ELS, UV-Vis spectroscopy, and TEM. The penetration ability of the nanoparticles through plant leaves was investigated using wheat sprouts. After 24 h of saturation, the plants were dried, and their leaves and seedlings were dissolved in nitric acid; the solution was analyzed by ICP-AES. HA-coated ZnO nanoparticles showed competitive performance compared to conventional zinc chelates used in agriculture. Wheat leaf studies confirmed that nanoparticle accumulation and uptake are pH-dependent, with acidic conditions favoring HA-coated ZnO nanoparticles. These findings provide valuable insights for developing advanced zinc-containing formulations for foliar crop fertilization. The demonstrated effectiveness of HA-stabilized ZnO nanoparticles suggests they represent a promising alternative to traditional zinc supplements in agriculture.

## 1. Introduction

Zinc serves as a crucial micronutrient in agricultural systems, playing indispensable roles in plant physiology and fertilizer formulations. However, widespread zinc deficiency in soils has become one of the most common micronutrient limitations worldwide, which not only reduces crop productivity but also diminishes the nutritional quality of harvests, ultimately compromising animal and human health through the food chain [1,2]. This essential trace element takes part in critical physiological processes, including enzyme synthesis (e.g., dehydrogenases and superoxide dismutases), growth regulation, chlorophyll formation, photosynthesis and carbohydrate metabolism. It also enhances plant stress resistance to drought, diseases, and low temperatures. Among various zinc forms in agrochemistry, zinc oxide offers distinct advantages due to its high zinc concentration (~80% Zn), making it economically helpful; ZnO does not dissolve quickly in water, which reduces leaching, and provides a prolonged release effect, ensuring slow zinc delivery to plants [3,4,5,6,7]. The latter is confirmed by the summary of the results described in Refs. [8,9,10,11,12], where Zn-chelate, zinc sulfate, and ZnO nanoparticles were tested; we can compare these zinc fertilizer sources by general parameters (Table 1).

Earlier findings indicate that ZnO nanoparticles improve photosynthetic efficiency, increase chlorophyll content, stabilize stomatal aperture, and reduce production of reactive oxygen species [13].

Humic substances are natural macromolecules that are essential for sustainable agriculture, offering a natural solution to improve productivity, soil health, and environmental resilience. Their use aligns with organic farming, precision agriculture, and climate-smart practices, making them promising biostimulants and soil amendments in modern agronomy [14,15,16]. Due to their amphiphilic nature, humic substances can act as surfactants and therefore influence the stability and behavior of nanoparticles. The addition of humic substances into the reaction mixture during ZnO nanoparticle synthesis results in a significant decrease in the particle size [17]. The presence of different functional groups in the humic acids’ composition provides their high affinity for binding heavy metals, especially cations of zinc and cadmium, promoting their leaching from soils [18,19,20,21]. Since the colloidal behavior of humic substances is controlled by ionic strength, Zn^2+^ and humic acids will have a mutual influence on each other’s self-assembly. Note that the behavior of nanoparticles in the presence of humic substances that is described in the literature is contradictory [22,23,24]. In particular, the doping of ZnO nanoparticles with HA has been shown to enhance their photocatalytic activity [25,26]. This is evidenced by the generation of reactive oxygen species by the hybrid particles, both under irradiation and in the dark, which distinguishes them from free ZnO. However, the effect of HA on ZnO nanoparticles is strongly dependent on environmental factors, including concentration, pH, and ionic strength. Bian et al. investigated the impact of HA on the smallest ZnO species, approximately 4 nm in diameter [27]. They reported that at low concentrations, HA induces charge neutralization, leading to nanoparticle aggregation, whereas at high concentrations, it stabilizes the suspension through steric hindrance. Moreover, HA promotes ZnO dissolution at high pH via ligand-promoted mechanisms, while exerting no significant effect under acidic conditions. The latter finding is particularly relevant for assessing the bioavailability of ZnO nanoparticles.

The present study addresses two critical objectives: (1) synthesis of HA-stabilized ZnO nanoparticles, and (2) assessment of nanoparticle efficacy as zinc sources for foliar fertilization. The key novelty of this work lies in the comparative evaluation of ZnO synthesized in the presence of HA, ZnO physically mixed with HA, ionic Zn^2+^, and a commercial chelate under varying pH conditions.

To solve this issue, ZnO nanoparticles were synthesized from zinc acetate in an aqueous solution and in the presence of humic acids. The leaves of wheat seedlings were treated with aqueous solutions containing either zinc cations in the presence of humic acids or zinc oxide nanoparticles, both with and without humic acid additives.

## 2. Materials and Methods

### 2.1. Materials

Sakhalin sodium humate (Zeleny Ostrov, Shakhtersk, Russia) with a humic acid content of ca. 80% was used. The detailed description of HA was provided previously [28,29]. In the present study, we additionally characterize the sample by FTIR. The results and the methodological details are presented in the corresponding sections of the paper. Acetate buffers of pH 8.0 and 5.0 were prepared by the addition of ammonia or acetic acid to 0.05 M ammonium acetate. Both components were purchased from Reakhim (Moscow, Russia) and used as received. Zinc acetate dihydrate (Reakhim, Moscow, Russia) and urea (Reakhim, Moscow, Russia) were used without further purification. For the control experiment, Zinc Chelate Fertilizer (ULTRAMAG, Schelkovo, Russia) was used.

### 2.2. Methods

#### 2.2.1. Synthesis of ZnO and ZnO-HA Nanoparticles

Synthesis of ZnO nanoparticles was performed according to a modified procedure described in reference [17]. A 2 M NaOH solution was added dropwise to 30 mL of 5.5 g/L Zn(CH_3_CO_2_)_2_·2H_2_O solution until the pH reached 12. The reaction mixture also contained 7.56 g/L HA when ZnO-HA nanoparticles were synthesized.

The reaction mixture was stirred in a shaker for 24 h at room temperature. The supernatant was collected from the precipitate after centrifugation. The precipitate was washed with water until it reached a neutral pH and dried at 60 °C to a constant weight.

#### 2.2.2. Nanoparticles Characterization

ZnO and ZnO-HA nanoparticles were analyzed using transmission electron microscopy at the Central Research Laboratory of Lomonosov Moscow State University.

X-ray diffraction analysis was performed using a Rigaku D/MAX 2500 diffractometer (Tokyo, Japan) equipped with a rotating anode (CuKα radiation, graphite monochromator). The scanning range was from 10 to 80° (2*θ* scale). Experimental data were processed using the STOE WinXPow software package (Tübingen, Germany) [30]. The interplane distances ($d_{hkl}$) were calculated using the Bragg-Wolfe equation (Equation (1)). The unit cell parameters were found by Equation (2) using the least squares method.

(1) $$n\lambda =2d_{hkl}\sin\;\theta$$

(2) $$\frac{1}{d_{hkl}^{2}}=\frac{4}{3}\left(\frac{h^{2}+hk+k^{2}}{a^{2}}\right)+\frac{l^{2}}{c^{2}}$$

Here λ is the X-ray wavelength (Cu-Kα ≈ 1.5406 Å), and θ is the angle between the incident X-ray beam and the crystallographic plane. Constructive interference occurs when the path-length difference between reflected X-rays is an integer multiple n of the wavelength λ. The orientation of a specific set of atomic planes is defined by its Miller indices (h,k,l), and the interplanar spacing is denoted by $d_{hkl}$.

The average crystallite size ($d_{XRD}$), or coherently scattering domain size, was estimated using the Scherrer formula:

(3) $$d_{XRD}=\frac{0.9\lambda }{\beta cos\;\theta }$$

Here 0.9 is the shape factor for spherical particles, *β* is the line broadening at half the maximum intensity (FWHM).

The average hydrodynamic size and electrokinetic potential (ζ-potential) of ZnO and ZnO-HA nanoparticles were determined using dynamic and electrophoretic light scattering (DLS and ELS, respectively) with a Malvern Zetasizer Nano ZS instrument (Malvern Instruments Ltd., Malvern, UK). The measurements were performed in triplicate, with a 60 s interval between each of them. The measurement results were processed using Malvern Instruments software (Worcestershire, UK). Aqueous ZnO suspensions with a concentration of 0.5 g/L were prepared for the investigation. Measurements were conducted with a detection angle of 173°. All measurements in this study were taken at a temperature of 25 °C. At least three measurements of each sample were taken to check for result repeatability. The intensity size distributions were obtained from the analysis of the correlation functions using the Multiple Narrow Modes algorithm in the instrument software [31].

The absorption spectra of colloidal solutions of ZnO and ZnO-HA nanoparticles were measured over the wavelength range of 200–700 nm in standard quartz cuvettes with an optical path length of l = 1 cm using a U-5100 spectrophotometer (Hitachi, Tokyo, Japan). The band gap energy ($E_{g}$) of ZnO and ZnO-HA nanoparticles was estimated using Equation (4) [32]:

(4) $$E_{g}=\frac{hc}{\lambda_{max}}$$

Here h is the Planck constant is equal to 6.626 × 10^−34^ J·s, c is the speed of light in vacuum is equal to 299,792,458 m/s, $\lambda_{max}$ is the wavelength of the exciton absorption peak.

Another method for determining $E_{g}$ is the Tauc plot method, based on Equation (5) [33]:

(5) $$(\alpha h\nu )=B{\left(h\nu -E_{g}\right)}^{n}$$

Here *α* is the absorption coefficient, *ν* is the frequency of the incident radiation, *B* is the constant, *n* = ½ is the constant determined by the type of electronic transition (here, the direct allowed transition). The absorption coefficient can be determined from Equation (6):

(6) $$\alpha =\frac{2.303A}{l}$$

Here, A is the absorbance obtained from the UV-visible spectrum; l = 1 cm is the optical path length or the width of the quartz cuvette. The *E*_g_ value was determined by extrapolating the linear region of the Tauc plot to zero ((*αhν*)^2^ = 0). The value obtained from the *hν*-axis corresponds to *E*_g_.

The registration of ATR spectra with Fourier transform was carried out using a Vertex 70 (Bruker Optik GmbH, Ettlingen, Germany) with a resolution of 2 cm^−1^, 64/64 sample/background scans in the IR region from 100 to 4000 cm^−1^ using a MIRacle single-reflection ATR attachment with a diamond crystal (Pike Instr., Madison, WI, USA). Every measurement was taken at room temperature.

#### 2.2.3. Germination of Wheat Sprouts

Zinc uptake was studied using common wheat *Triticum aestivum* L. var. Akhtyrchanka. In these experiments, 14-day wheat seedlings were used. The seeds were germinated in the dark at 24 °C for 24 h. Then, the imbibed seeds with the visible germinal root were transferred into 1 L polyethylene tanks containing perlite supported with Knop nutrient solution (0.14 g/L KH_2_PO_4_, 0.1 g/L KCl, 0.14 g/L KNO_3_, 1.42 g/L MgSO_4_·7H_2_O, 4.88 g/L Ca(NO_3_)_2_·12H_2_O, pH 5.5) and cultivated in a growth chamber (12 h light/12 h dark photoperiod, illumination 200 μmol m^−2^ s^−1^; 24 °C) for 13 d. Spray solutions were prepared in phosphate buffer with the addition of 1 M urea.

#### 2.2.4. Studying Zinc Uptake by Leaves of Wheat Sprouts

Acetate buffers of pH 5 and 8 were used. ZnO was used at pH 8 in free form and in the mixture with HA (Sakhalin sodium humate). ZnO-HA was used at pH 5 and 8. Two control systems were used. (1) Plants that grew on the same medium were not immersed in any solution. (2) Commercially available Zinc Chelate Fertilizer that is a premium micronutrient formulation designed and recommended to enhance plant vitality and prevent chlorotic leaf spotting. Three plants were used for each solution.

The upper part of the leaf (1 cm) was immersed in 1 mL of the solution for 24 h. Then it was removed from the solution, washed in water, wiped, and dried to a constant weight. A plant was cut into pieces (a submerged leaf, another leaf, and a stem). Each fragment was weighed and then dissolved in concentrated nitric acid during boiling. The zinc content in the solutions was determined by ICP-AES using an Agilent 720 ICP-AES spectrometer with an axial view (Agilent, Mulgrave, Australia) [34].

### 2.3. Statistical Analysis

Mean values and standard deviations were calculated using OriginPro and Statistica 10 software [35,36]. When zinc uptake by wheat seedlings was studied, the non-parametric Kruskal-Wallis test was applied, followed by Dunn’s post hoc test for multiple comparisons when appropriate.

## 3. Results and Discussions

### 3.1. ZnO and ZnO-HA Nanoparticles Characterization

To prove the reproducibility of the techniques, each synthesis was repeated three times. The yield of zinc oxide without humic acids was 92 ± 2%. It is impossible to estimate the yield at this stage for a sample synthesized in the presence of HA, since HA is present in the brown precipitate.

**Figure 1:**
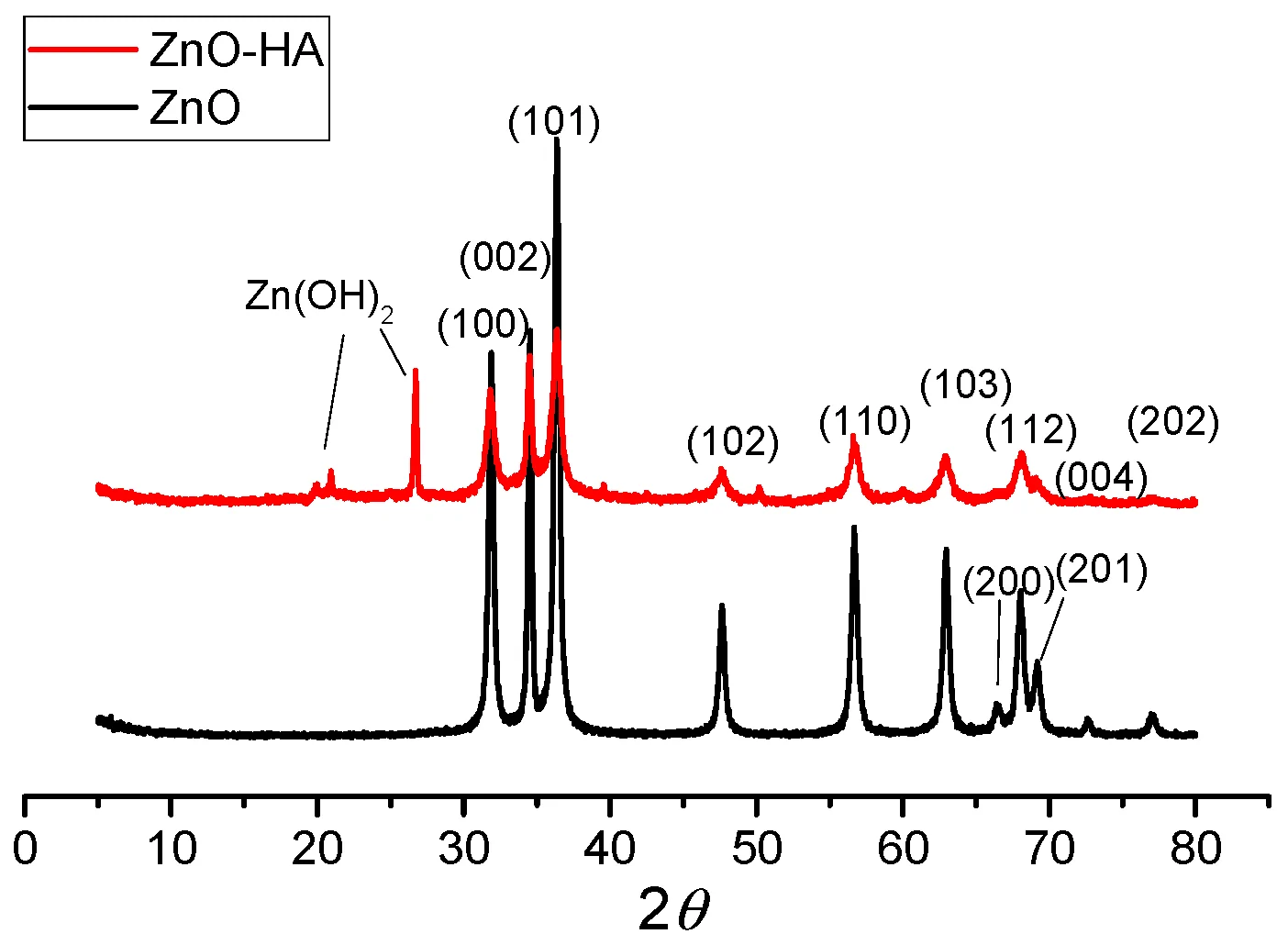
XRD of ZnO and ZnO-HA samples.

The XRD of both samples is shown in Figure 1. In the sample obtained without HA, all observed diffraction peaks: (100), (002), (101), (102), (110), (103), (200), (112) and (201) correspond to the single—phase hexagonal structure of ZnO wurtzite (COD ID 1011259, Crystallography Open Database) [37]. The presence of other phases in this case has not been revealed. The interplane distances (*d_hkl_*) were calculated using the Bragg-Wolfe equation (Equation (1)). The unit cell parameters were found by Equation (2) using the least squares method.

The orientation of a specific set of atomic planes is defined by its Miller indices (*h, k, l*), and the interplanar spacing is denoted by d. The following lattice parameters were obtained: a = b = 3.2414 ± 0.0002 Å, c = 5.1934 ± 0.0002 Å, α = β = 90°, γ = 120°.

In the sample synthesized in the presence of HA, a zinc hydroxide phase was detected, accounting for approximately 18 wt.% of the material [17]. This can be explained because the humic matrix, within which ZnO nanoparticles nucleate and grow, creates diffusion barriers to the migration of ionic components of the reaction system (OH-ions). These barriers include a steric effect arising from the high molecular weight of HA and an electrostatic effect associated with deprotonated HA macromolecules. As a result, Zn(OH)_2_ formed during the reactions does not have sufficient time to convert into a water-soluble hydroxocomplex, and HA captures and stabilizes this compound. A similar interaction mechanism also occurs during the formation of feroxyhyte nanoparticles [38].

The broadening of the signals is probably due to a decrease in the size of the nanoparticles. The average crystallite sizes calculated from the broadening of the diffraction peaks are 20 ± 1 nm and 11 ± 1 nm for ZnO and ZnO-HA nanoparticles, respectively. These data indicate that ZnO-HA nanoparticles are smaller than ZnO nanoparticles. This is likely because HA blocks the growth facets of crystallites during synthesis, thereby stabilizing smaller particles [17].

**Figure 2:**
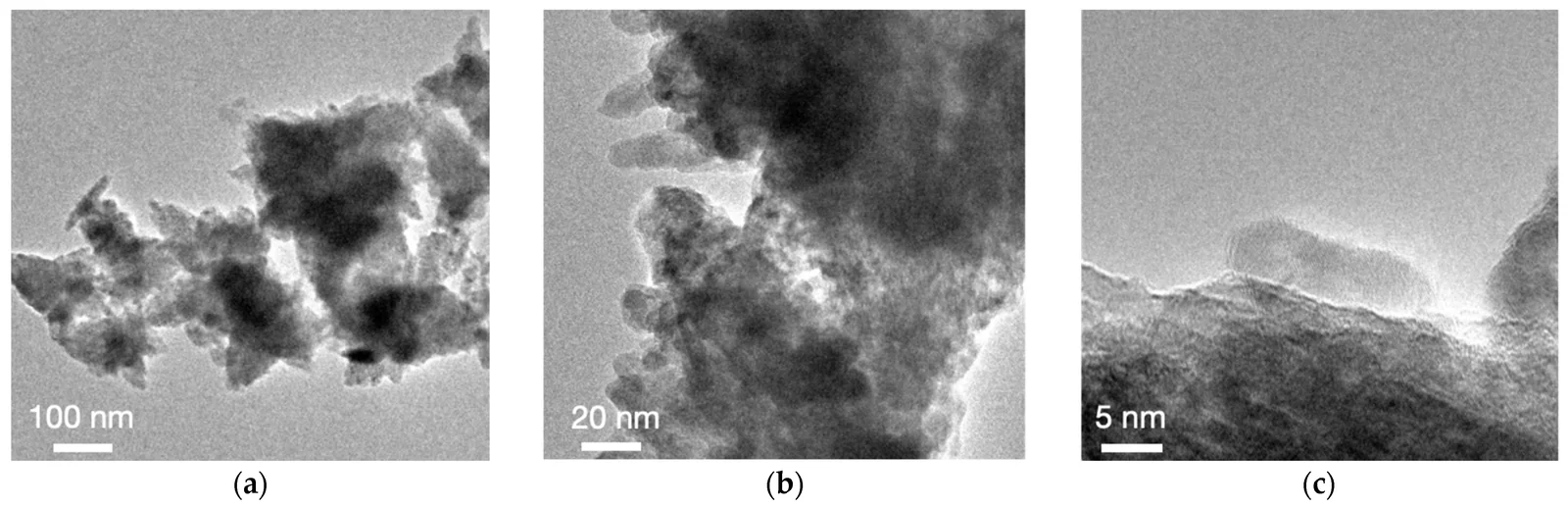
TEM images of ZnO nanoparticles obtained at scale bars (**a**) 100 nm, (**b**) 20 nm, and (**c**) 5 nm.

Figure 2 shows TEM images of ZnO nanoparticles.

TEM analysis revealed that most ZnO particles exhibited distinct elongation. However, in this case, the determination of the average particle size is challenging because the sample contains very few clearly distinguishable individual particles, which complicates data processing. The determined interplane distances being close to 2.8 Å confirm the hexagonal structure of ZnO wurtzite.

**Figure 3:**
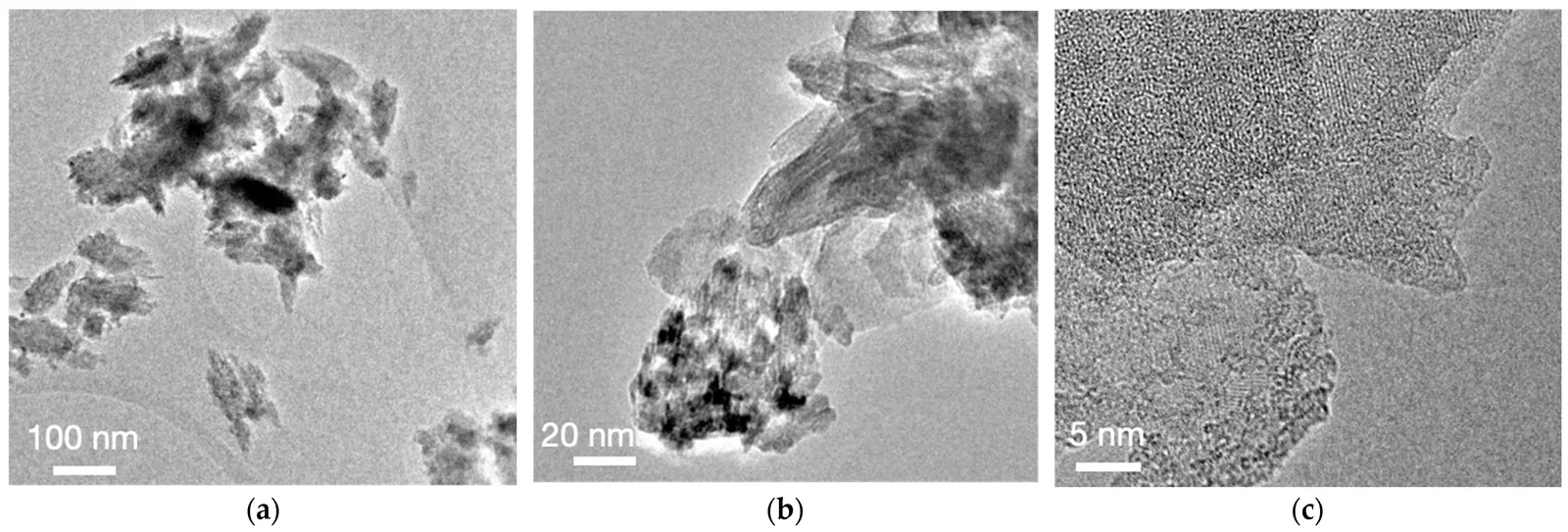
TEM images of ZnO nanoparticles synthesized in presence of HA, obtained at scale bars of (**a**) 100 nm, (**b**) 20 nm, and (**c**) 5 nm.

Figure 3 shows TEM images of ZnO nanoparticles that were synthesized in the presence of HA.

The presence of HA during zinc oxide synthesis resulted in plate-like nanoparticle formation, as evidenced by the observed layered morphology. The average sizes of these particles are 121 ± 14 and 56 ± 6 nm, corresponding to the major and minor radii, respectively. Unlike the ZnO sample, the ZnO-HA sample contains a sufficient number of well-resolved individual particles to allow reliable size determination. This may be attributed to the presence of HA, which likely reduces particle-particle association through electrostatic interactions, resulting in a more loosely packed structure and better separation of individual particles. Note that ZnO nanoparticles synthesized in the presence of HA also possess a wurtzite structure, according to the determined interplane distances that were close to 2.8 Å.

**Figure 4:**
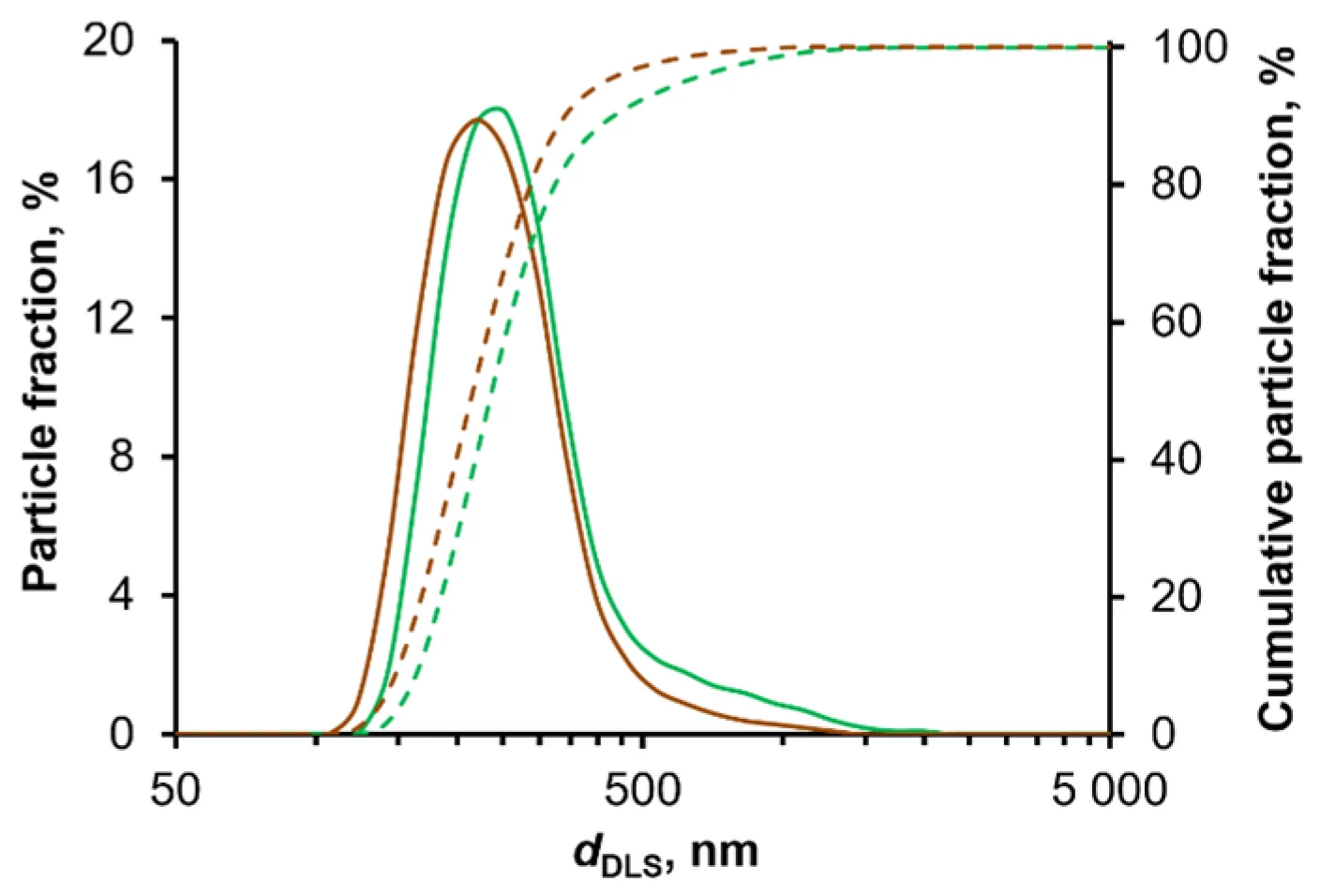
Differential (solid lines) and cumulative (dashed lines) hydrodynamic diameter (dDLS) distribution curves of ZnO nanoparticles. The green curves correspond to the ZnO sample, and the brown curves correspond to the ZnO-HA sample.

To compare the state and behavior of ZnO and ZnO-HA in the aqueous suspension, dynamic light scattering analysis was performed. Note that this method is not ideally suited for absolute size determination of aggregating nanoparticles and especially for particles of non-spherical shape. However, it can be applied for comparative analysis among samples measured under identical conditions. Given the aggregating nature and non-spherical morphology of the nanoparticles, only the Z-average hydrodynamic diameter and polydispersity index (PDI) were used for sample characterization and comparative analysis. Based on the results of the DLS measurements, differential and cumulative particle size distribution curves, obtained for aqueous nanoparticle suspensions at neutral pH, were plotted, as shown in Figure 4. The average hydrodynamic diameters calculated from the data obtained are 307 (PDI = 0.379) and 260 nm (PDI = 0.448) for the ZnO and ZnO-HA nanoparticles, respectively. It should be noted that the reported values fall outside the size range typically attributed to nano-objects (1–100 nm). This is related to the specific features of particle detection and size calculation in the DLS method. The hydrodynamic diameter is determined by the thickness of the electrical double layer formed at the solid/liquid interface. Therefore, the hydrodynamic diameter is always larger (higher) than the actual particle diameter. The slight decrease in nanoparticle size observed during synthesis in a HA medium is consistent with DLS data reported in the research work [26]. This effect is likely associated with the action of HA as a surfactant of polyelectrolyte nature. In water at neutral pH, HA is capable of stabilizing smaller particles. Thus, despite the presence of a HA shell, the average hydrodynamic diameter of ZnO-HA nanoparticles is smaller than the corresponding value for ZnO nanoparticles. For comparison, the z-average values, i.e., the average intensity-weighted hydrodynamic diameters, for ZnO and ZnO-HA nanoparticles are 772 and 622 nm, respectively. The key feature of this parameter is its sensitivity to particles of different sizes: large particles and aggregates scatter light disproportionately strongly, thereby shifting the z-average toward higher values. Based on the data obtained, it can be concluded that both systems contain larger particles and aggregates.

Since acetate buffer solutions were used in the subsequent plant experiments, the colloidal stability of ZnO and ZnO-HA nanoparticle suspensions was evaluated over time at pH 5 and 8. At pH 5, ZnO nanoparticles exhibited limited aggregation, likely due to the prevalence of dissolution over particle–particle interactions. In contrast, at pH 8, ZnO nanoparticles formed aggregates with a mean hydrodynamic diameter of approximately 450 nm. This aggregation is presumably driven by the ionic strength contributed by NH_4_^+^ and CH_3_COO^−^ ions from the buffer. After 24 h, sedimentation of the larger aggregates resulted in a redistribution of the remaining suspended fraction toward smaller sizes. For ZnO-HA suspensions, more pronounced aggregation was observed at both pH values, with mean diameters of 704 nm at pH 5 and 1148 nm at pH 8. After 24 h, sedimentation effects became dominant in both systems, analogous to the behavior observed for bare ZnO. Overall, these findings indicate that the presence of humic acid promotes aggregation of ZnO nanoparticles under the tested conditions, while acidic pH partially mitigates this effect through dissolution.

ζ-potential values, obtained for aqueous nanoparticle suspensions at neutral pH, are 29 (SD = 9) and −31 (SD = 5) mV for ZnO and ZnO-HA nanoparticles, respectively. The sign and magnitude of the ζ-potential of ZnO nanoparticles synthesized without HA are in good agreement with the literature data [39]. The ζ-potential of ZnO-HA nanoparticles becomes negative, while its absolute value remains close to that of ZnO nanoparticles. The negative sign of this parameter indicates the presence of a HA shell on the nanoparticle surface. At neutral pH, the functional groups of HA, -OH and -COOH groups, are partially deprotonated. Therefore, the surface charge is negative [39]. Similar behavior has also been observed, for instance, for silver nanoparticles [40] and iron oxide nanoparticles [41] synthesized in a HA medium. Partially deprotonated HA polyanions form an electrostatic barrier that hinders, but, according to the DLS data, does not completely prevent particle aggregation in solution. The moderate colloidal stability of aqueous suspensions of ZnO-HA nanoparticles may also be explained by the steric effect of surface-bound HA.

Application of acetate buffers reduces the ζ-potential of ZnO to values close to 6 at pH 5 and to 18 (SD = 12) mV at pH 8. In the case of ZnO-HA, the ζ-potential value was reduced to −17 (SD = 20) mV. The reason for the last observation is the possible shielding effect of the acetate buffer neutralizes any potential differences that might arise due to differences in pH. This fact also confirms the presence of HA on the surface of nanoparticles.

**Figure 5:**
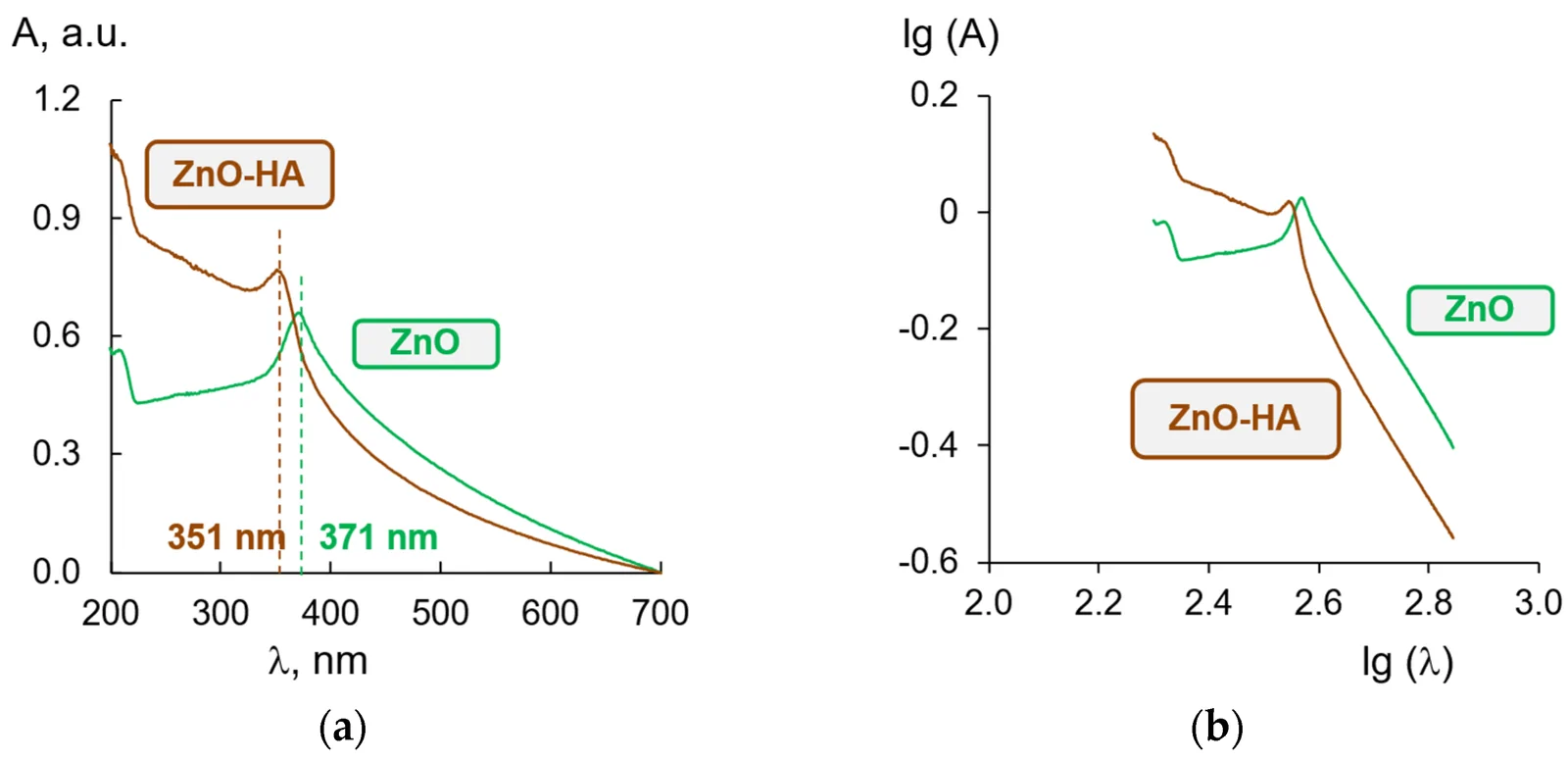
Absorption spectra of colloidal solutions containing ZnO (green curve) and ZnO-HA nanoparticles (brown curve): (**a**) linear-scale plot; (**b**) log–log plot.

The optical properties of ZnO and ZnO-HA nanoparticles were investigated using UV-visible spectroscopy. The UV-visible absorption spectra of the samples are shown in Figure 5. The spectra exhibit absorption maxima at wavelengths of 371 nm (ZnO) and 351 nm (ZnO-HA). The exciton peak located in this wavelength region is characteristic of ZnO semiconductor [42]. In the case of nanoparticles synthesized in an HA medium, the absorption maximum is shifted toward shorter wavelengths, i.e., a so-called blue shift is observed. This, presumably, indicates a decrease in the size of ZnO crystallites due to the quantum confinement effect in nanoparticles [33]. This result correlates with the nanoparticle size data obtained by XRD analysis. The observed shift in the absorption wavelength may also be influenced by additional factors, including nanoparticle morphology and the relative contents of the Zn(OH)_2_ phase and HA in the ZnO-HA sample. For instance, nanoparticles with flatter morphology may shift the exciton peak toward shorter wavelengths [43,44,45]. The influence of the Zn(OH)_2_ phase was reported in [46]; however, the magnitude of the shift was only 4 nm. HA may also affect the position of the absorption maximum; however, clear evidence for such an effect is lacking in the literature. The position of the absorption maximum is likely to remain unchanged when the absorption contribution of the humic component is considered, as typical HA absorption spectra exhibit no peak near 350 nm. The overall shape of the absorption spectrum of ZnO-HA nanoparticles also remains essentially unchanged. On the other hand, HA may potentially increase $E_{g}$, as discussed below. In addition, the absorption spectrum of the ZnO-HA nanoparticle suspension shows an increase in absorbance at wavelengths of 322 nm and below, which is associated with radiation absorption by aromatic fragments of HA. This may again indirectly indicate the presence of a humic shell on the nanoparticle surface.

**Figure 6:**
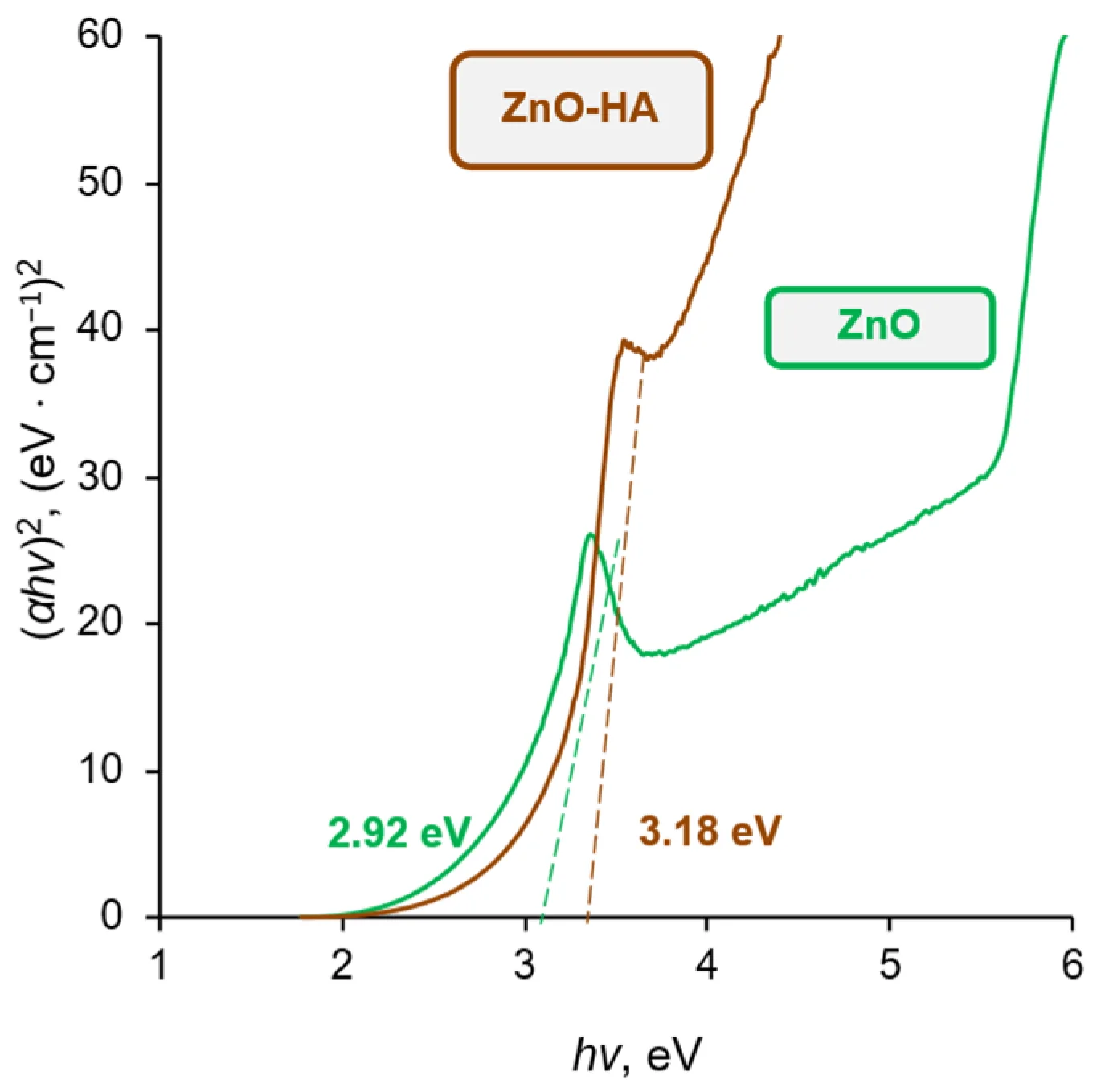
Tauc plots of ZnO (green curve) and ZnO-HA nanoparticles (brown curve).

The band gap values obtained from Equation (4) are 3.35 and 3.54 eV for ZnO and ZnO-HA nanoparticles, respectively. The Tauc plots shown in Figure 6 give $E_{g}$ of 2.92 and 3.18 eV, respectively. Accounting for HA absorption has a negligible effect on the values of these parameters. The increase in this parameter by 0.19 eV, presumably, also indicates a decrease in the size of ZnO crystallites [43]. The observed trends in the optical properties and size characteristics of ZnO and ZnO-HA nanoparticles are in good agreement with previous studies devoted to humic composites based on ZnO nanoparticles [17,39]. Moreover, the increase in $E_{g}$ observed for ZnO-HA nanoparticles may be associated with the Burstein-Moss effect. HA, acting as a dopant, contains electron-rich aromatic structures that may increase the concentration of conduction-band electrons, thereby shifting the optical absorption edge of ZnO toward higher energies. A similar effect has been reported, for instance, for cerium dioxide nanoparticles synthesized in the presence of lignin (a humic-like substance) [47].

However, the methods mentioned above are insufficient to demonstrate the coating. Furthermore, it is the surface of nanoparticles that determines their properties and behavior in complex systems [48]. Therefore, we have detected ATR-FTIR spectra of bare ZnO nanoparticles, pristine humic acids, and ZnO oxide coated by the HA (Figures S1–S5).

The band assignment is summarized in Table 2. The following observations indicate an oxide coating. First, organic bands are present in the composite and are absent from bare ZnO. The composite shows a carboxylate pair at 1577 and 1372 cm^−1^. Bare ZnO also has weak bands in this window, but with a clearly different four-band pattern (1554, 1504, 1418, and 1389 cm^−1^) characteristic of surface carbonate/adventitious species on ZnO, not of a carboxylate doublet. Second, the free carboxylic C=O feature is lost. The 1698 cm^−1^ shoulder of protonated -COOH, clearly resolved in the parent HA, is not detectable in the composite. This is the classic signature that the carboxyl groups involved in the interface are deprotonated and coordinated rather than free [49,50]. Finally, the carboxylate splitting changes on binding. In the parent HA, ν_as_(COO^−^) is observed at 1557 cm^−1^ and ν_s_(COO^−^) is observed at 1370 cm^−1^, i.e., Δν = 187 cm^−1^, typical of largely ionic/free carboxylate in humic material. In the composite, νas shifts to 1577 cm^−1^ (+20 cm^−1^) while νs is essentially unchanged (1372 cm^−1^), giving a tremendous shift Δν = 205 cm^−1^. Following the Deacon-Phillips correlation [49], an increase in νas and in Δν relative to the free/ionic form indicates monodentate (or strongly asymmetric) coordination of one carboxylate oxygen to a metal center, rather than physical admixture, bidentate chelation, or bridging. This shift cannot be replicated by any linear combination of the two parent spectra, thus indicating chemical bonding at the ZnO surface.

Thus, the combination of the analytical methods employed confirms the presence of humic substances bound to the surface of the zinc oxide nanoparticles. This sample was used in the next stage to assess the uptake of zinc in various forms (ions, nanoparticles, chelate) by plants.

### 3.2. Zinc Uptake by Leaves of Wheat Sprouts

Zinc uptake by leaves of wheat sprouts was determined by a controlled leaf-immersion assay. Table 3 shows zinc accumulation in the leaves and stems of wheat. Note that here we consider only a leaf that had no contact with the solution. Therefore, we are specifically discussing the uptake of zinc by the plant. Differences between groups were assessed using the Kruskal–Wallis test (*p* < 0.001), followed by a post hoc Dunn’s test and Bonferroni correction. Groups that do not share common letters differ statistically significantly (*p* < 0.05).

Both types of ZnO nanoparticles and zinc in the form of Zn^2+^ as a component of the solution were used in the analysis of zinc distribution in the plant’s organs during foliar uptake. Zinc content in the initial solutions and in the plant’s organs was analyzed by ICP-AES. It has to be emphasized that ICP-AES measurements provide total zinc concentration and cannot distinguish between uptake of intact ZnO nanoparticles, dissolved Zn^2+^ ions, Zn–HA complexes, or other Zn-containing species.

Table 3 also illustrates zinc binding in plant organs as a ratio between its concentration in the organ normalized to the concentration in the solution:

(7) $$k_{d}^{organ}=\frac{c_{organ}}{c_{solution}}$$

It was observed that with the commercially available “Zinc Chelate”, zinc is concentrated in the leaves compared to the control, whereas it is not concentrated in the stem. It is difficult to draw conclusions about the stem because of the high zinc content in the stem of the control group. However, the calculated distribution constants for Zn(II), ZnO, and ZnO-HA are higher than those for Zinc Chelate. In the cases of ZnO mixed with HA and Zinc Chelate, the values of the distribution constants are comparable. According to this parameter, the systems we are considering are comparable to the commercial sample. Since our study used only a single zinc concentration in an acetate buffer at two pH values, and other parameters were kept constant, we cannot apply the concept of equivalent Zn doses. Therefore, for the comparative assessment analysis, we will use the introduced distribution constant, as it takes into account the correction for the zinc concentration in the saturating solution.

Let us now consider the concentration in the leaves. As it follows from Table 3, the zinc uptake is controlled by pH. At pH 8, the concentration of zinc in leaves for all tested samples was statistically comparable to the control group and lower than in a commercially available zinc-containing sample. The same applies to the distribution constant.

At pH 5, both ZnO synthesized in the presence of HA and mixed with them shows higher content in leaves compared with the control group. Nevertheless, the distribution constant for ZnO synthesized in HA solution shows a higher value compared with the commercial sample.

Thus, ZnO synthesized in the presence of HA and applied at pH 5 showed performance comparable to that of the commercial product, making it a promising candidate for further investigation as an agrochemical.

## 4. Conclusions

In summary, this study shows that humic acid-coated ZnO nanoparticles exhibit a distinct morphology and demonstrate uptake in wheat leaves comparable to that of the commercially available zinc chelate, with a pH-dependent accumulation pattern that suggests potential for optimizing zinc-based agrochemical delivery. These findings position HA-modified ZnO nanoparticles as a subject of interest for sustainable agriculture, warranting further exploration alongside conventional formulations. Future research should extend uptake studies to a broader range of crop species and investigate the physiological effects of these zinc forms.

## Figures and Tables

**Table 1:** Comparative analysis of zinc fertilizer sources by key parameters.

Parameter	Zinc Sulfate	Zn-Chelate	ZnO-Nanoparticles	Ref.
Solubility	Very high solubility in water and rapid release of Zn^2+^ ions	High solubility	Very low water solubility and slow dissolution over time	[8,11,12]
Soil behavior	High leaching losses. Leaching is strongly pH-dependent	High leaching of chelates of applied zinc, especially in acidic and calcareous soils	Low leaching losses	[12]
Plant Zn uptake	High zinc accumulation in roots and shoots	High zinc uptake efficiency	Lower Zn uptake compared to ZnSO_4_ in most cases	[8,9,10]
Oxidative stress response	Induces strong antioxidant enzyme activity as a defense response.	Positive effect on antioxidant enzymes. Overall lower oxidative damage compared to ZnSO_4_.	Lower oxidative stress compared to ZnSO_4_.	[8,9,10]
Chlorophyll and photosynthetic pigments	Increases chlorophyll at concentrations 100–200 mg/kg. High concentrations reduce chlorophyll and carotenoid content.	Improves total chlorophyll and carotenoid content, but less effective than ZnO nanoparticles.	Highest increase in total chlorophyll and carotenoids.	[8,9,10]
Environmental risk	High leaching potential results in risk of groundwater contamination.	High environmental risk due to excessive leaching in both acidic and calcareous soils.	Lowest environmental risk due to minimal leaching	[12]

**Table 2:** Band assignment for HA, bare ZnO and the ZnO-HA composite.

Substances			Possible Band Assignment	Comment	Ref.
HA	ZnO	ZnO-HA			
2919, 2851	–	–	ν_as_/ν_s_ (CH_2_), ν(CH_3_) aliphatic backbone	Organic C is present in composite as a thin layer	[51,52]
1698 (shoulder)	–	–	ν(C=O) of protonated -COOH	Carboxyls deprotonated/coordinated	[49,50]
1662	1636	–	conjugated/quinone C=O, amide I; δ(H–O–H) of adsorbed water on ZnO	–	[50,53]
1559	1549, 1502	1587	ν_as_(COO^−^) + aromatic C=C; on bare ZnO: surface carbonate	+20 cm^−1^ shift indicates coordination	[49,50,53]
1365	1389	1377	ν_s_(COO^−^), δ(O–H) phenolic, δ(CH–)	Position unchanged as the whole Δν change is carried by ν_as_, as expected for monodentate binding	[48,52,53]
1278, 1248	–	–	ν(C–O) of aryl ethers/phenols and of -COOH	–	[50,53]
936, 912	–	937, 913	δ(Al–Al–OH), kaolinite octahedral sheet	Mineral matrix	[54]
–	833	–	δ(CO_3_^2−^) out-of-plane, surface carbonate on ZnO	ZnO suface species	[53,55]
796, 775	–	799, 777	ν_s_(Si–O–Si), α-quartz doublet	Marker of crystalline α-quartz	[56,57]
691	–	694	δ(Si–O–Si) symmetric bend, α-quartz	Confirms α-quartz	[55,56]
–	378	367	Zn–O	–	[58]

**Table 3:** Zinc concentration in plant organ (mg per kg of tissue) and zinc binding ($k_{d}^{organ}$) in leaves and stem. Zinc Chelate is a commercially available agrichemical, ZnO-HA is nanoparticles that were synthesized in presence of HA, ZnO+HA is ZnO nanoparticles with addition of HA into the saturating solution, and Zn(II) is zinc in form of zinc acetate in buffer solution. Identical letters (A, B, C, D, E) indicate the absence of statistically significant differences between groups; different letters indicate significant differences.

pH	Sample	Zinc Concentration in Leaves		Zinc Concentration in Stems	
		Zinc Content, mg/kg	$k_{d}^{leaf}$	Zinc Content, mg/kg	$k_{d}^{stem}$
No solution	control	78.01 ± 10.34 ^C^	-	129.65 ± 5.87 ^AB^	-
pH 6.0 (measured value)	Zinc Chelate	171.64 ± 5.46 ^A^	140 ± 4 ^C^	94.17 ± 2.56 ^BC^	77 ± 2 ^E^
pH 5	ZnO-HA	123.01 ± 3.47 ^AB^	253 ± 7 ^A^	72.57 ± 2.18 ^C^	149 ± 4 ^BC^
	ZnO+HA	106.24 ± 3.40 ^BC^	119 ± 4 ^D^	83.00 ± 2.82 ^C^	93 ± 3 ^DE^
	Zn(II) pH	80.24 ± 2.50 ^C^	74 ± 2 ^E^	127.81 ± 4.36 ^AB^	118 ± 4 ^CD^
pH 8	ZnO-HA pH	74.09 ± 2.69 ^C^	191 ± 7 ^B^	66.11 ± 2.07 ^C^	170 ± 5 ^AB^
	ZnO+HA pH	105.79 ± 4.11 ^BC^	105 ± 4 ^DE^	102.30 ± 2.50 ^BC^	102 ± 3 ^DE^
	ZnO pH	82.53 ± 2.61 ^C^	100 ± 3 ^DE^	110.98 ± 3.93 ^BC^	134 ± 5 ^BC^
	Zn(II) pH	116.92 ± 3.29 ^ABC^	110 ± 3 ^DE^	182.90 ± 6.41 ^A^	172 ± 6 ^A^

## Data Availability

The original contributions presented in the study are included in the article, and further inquiries can be directed to the corresponding author.

## Supplementary Materials

The following supporting information can be downloaded at: https://www.mdpi.com/article/10.3390/nano16181183/s1, ATR-FTIR spectra of humic substances, zinc oxide nanoparticles, and zinc oxide-coated humic substances Figure S1: ATR-FTIR spectra of samples: humic substances (black line), zinc oxide nanoparticles (red line), and zinc oxide-coated humic substances (blue line) in the 4000–100 cm^−1^ range; Figure S2: ATR-FTIR spectra of samples: humic substances (black line), zinc oxide nanoparticles (red line), and zinc oxide-coated humic substances (blue line) in the 4000–2000 cm^−1^ range; Figure S3: ATR-FTIR spectra of samples: humic substances (black line), zinc oxide nanoparticles (red line), and zinc oxide-coated humic substances (blue line) in the 2000–800 cm^−1^ range; Figure S4: ATR-FTIR spectra of samples: humic substances (black line), zinc oxide nanoparticles (red line), and zinc oxide-coated humic substances (blue line) in the 1800–800 cm^−1^ range, Figure S5: ATR-FTIR spectra of samples: humic substances (black line), zinc oxide nanoparticles (red line), and zinc oxide-coated humic substances (blue line) in the 800–100 cm^−1^ range.
