# Supplementary material for: Enhancing ZnO Efficiency: Humic Substances as Natural Carriers and Stabilizers for ZnO Nanoparticles for Sustainable Agriculture
Source: Nanomaterials (Basel). 2026 Sep 19;16(18):1183. doi: 10.3390/nano16181183 (PMC13610382; doi:10.3390/nano16181183)
Supplement: Supplementary file 1 [file nanomaterials-16-01183-s001.zip › nanomaterials-4535277-supplementary.pdf]

Supplementary Materials

Enhancing ZnO Efficiency: Humic Substances as Natural Carriers and Stabilizers for ZnO Nanoparticles for Sustainable Agriculture

Tianyi Shen, Gleb N. Trishkin, Maria G. Chernysheva, Natalia A. Kulikova, Ivan V. Mikheev, Grigorii R. Chermashentsev, Sergey V. Maksimov, Gennadii A. Badun

ATR-FTIR spectra of humic substances, zinc oxide nanoparticles, and zinc oxide-coated humic substances

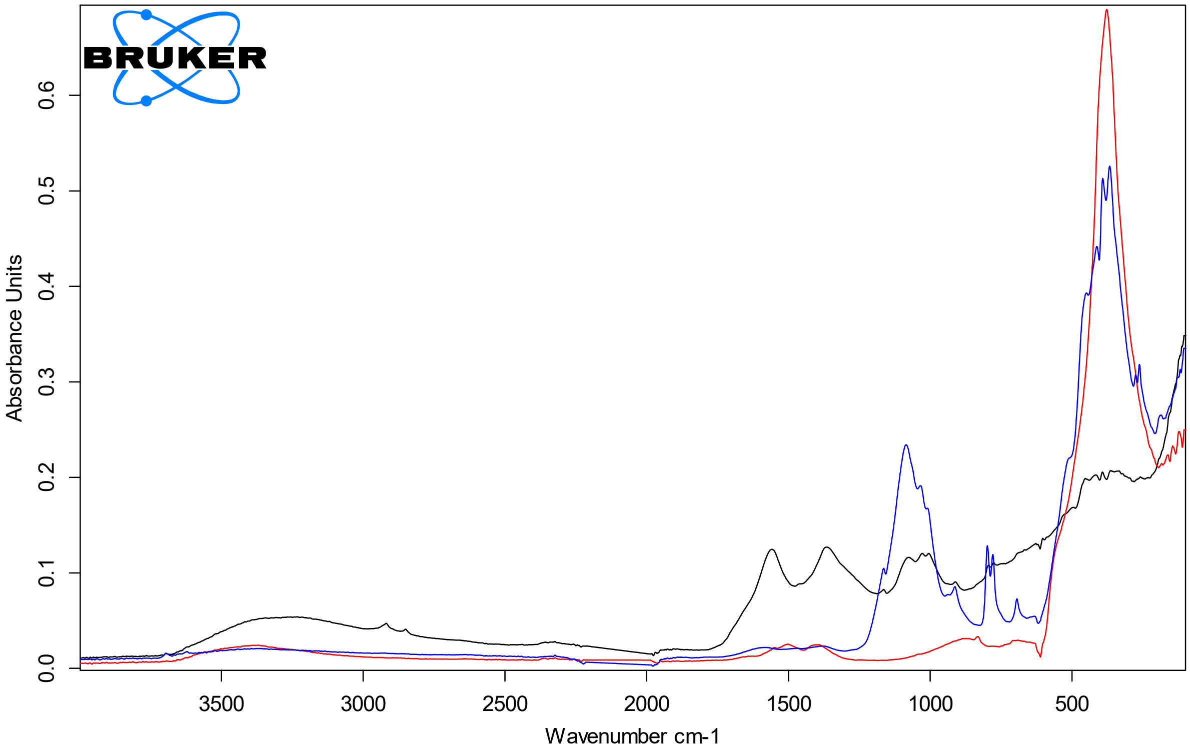

Figure S1. ATR-FTIR spectra of samples: humic substances (black line), zinc oxide nanoparticles (red line), and zinc oxide-coated humic substances (blue line) in the 4000-100 cm^{-1} range.

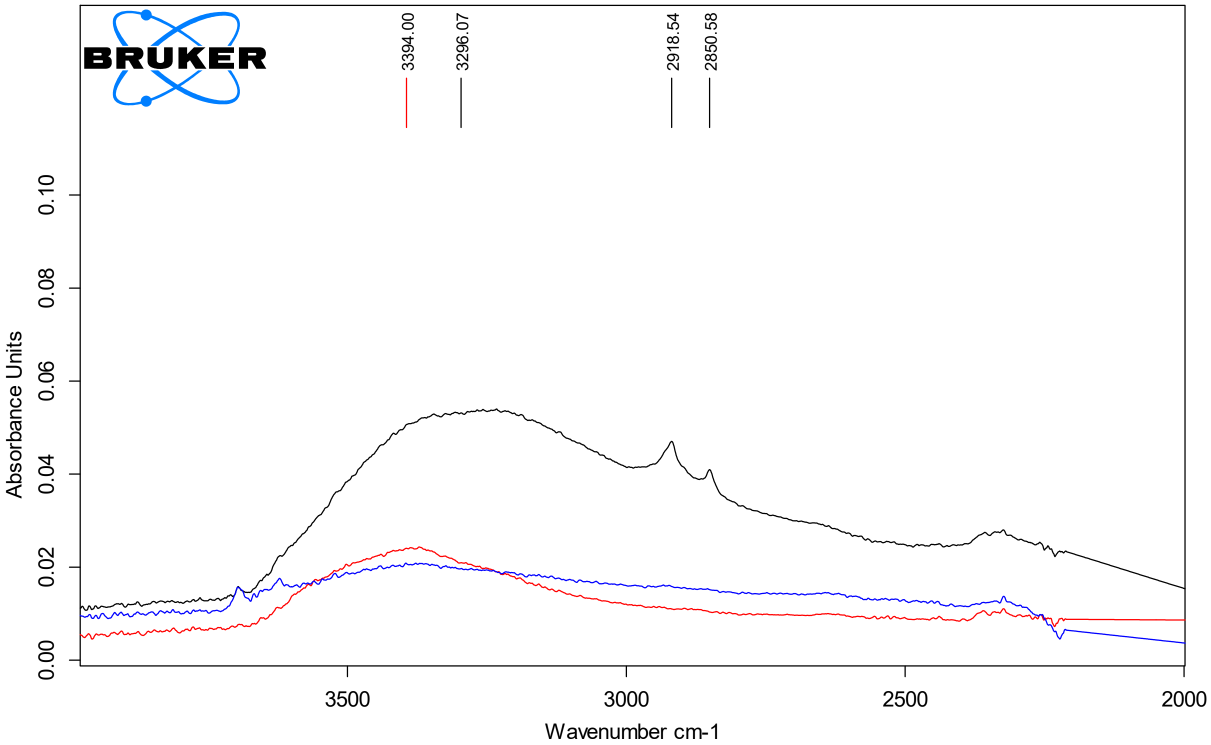

Figure S2. ATR-FTIR spectra of samples: humic substances (black line), zinc oxide nanoparticles (red line), and zinc oxide-coated humic substances (blue line) in the 4000-2000 cm^{-1} range.

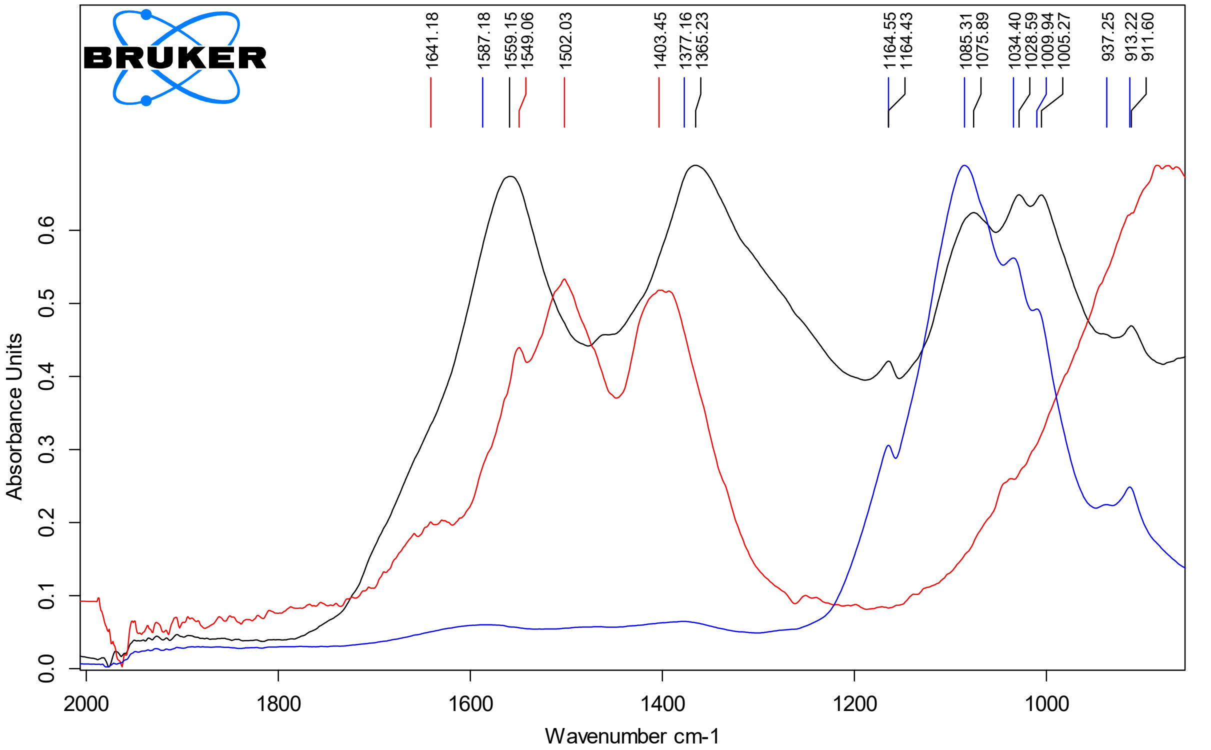

Figure S3. ATR-FTIR spectra of samples: humic substances (black line), zinc oxide nanoparticles (red line), and zinc oxide-coated humic substances (blue line) in the 2000–800 cm^{-1} range.

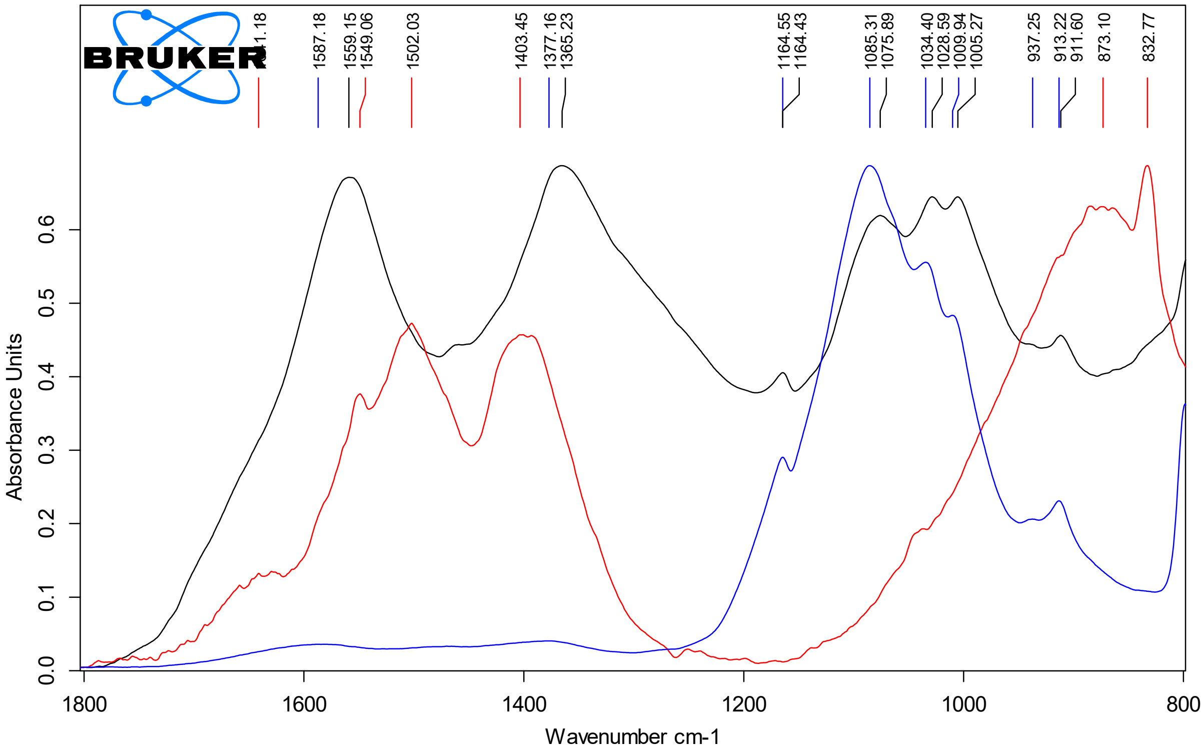

Figure S4. ATR-FTIR spectra of samples: humic substances (black line), zinc oxide nanoparticles (red line), and zinc oxide-coated humic substances (blue line) in the 1800–800 cm^{-1} range.

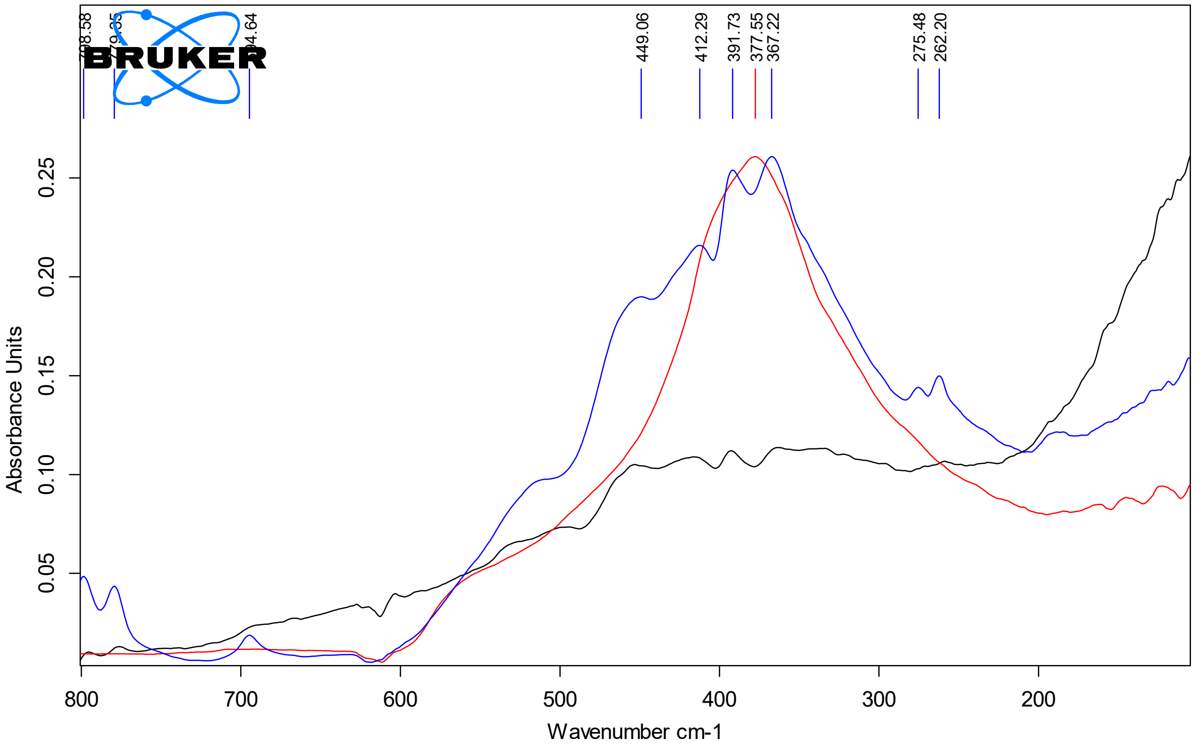

Figure S5. ATR-FTIR spectra of samples: humic substances (black line), zinc oxide nanoparticles (red line), and zinc oxide-coated humic substances (blue line) in the 800-100 cm^{-1} range.
